# Poly(ethylene glycol) and Cyclodextrin-Grafted Chitosan: From Methodologies to Preparation and Potential Biotechnological Applications

**DOI:** 10.3389/fchem.2017.00093

**Published:** 2017-11-07

**Authors:** Estefânia V. R. Campos, Jhones L. Oliveira, Leonardo F. Fraceto

**Affiliations:** ^1^Department of Environmental Engineering, Institute of Science and Technology, São Paulo State University, Sorocaba, Brazil; ^2^Department of Biochemistry and Tissue Biology, Institute of Biology, State University of Campinas, Campinas, Brazil

**Keywords:** chitosan, poly(ethylene glycol), grafting, copolymerization

## Abstract

Chitosan, a polyaminosaccharide obtained by alkaline deacetylation of chitin, possesses useful properties including biodegradability, biocompatibility, low toxicity, and good miscibility with other polymers. It is extensively used in many applications in biology, medicine, agriculture, environmental protection, and the food and pharmaceutical industries. The amino and hydroxyl groups present in the chitosan backbone provide positions for modifications that are influenced by factors such as the molecular weight, viscosity, and type of chitosan, as well as the reaction conditions. The modification of chitosan by chemical methods is of interest because the basic chitosan skeleton is not modified and the process results in new or improved properties of the material. Among the chitosan derivatives, cyclodextrin-grafted chitosan and poly(ethylene glycol)-grafted chitosan are excellent candidates for a range of biomedical, environmental decontamination, and industrial purposes. This work discusses modifications including chitosan with attached cyclodextrin and poly(ethylene glycol), and the main applications of these chitosan derivatives in the biomedical field.

## Introduction

**Figure 1:**
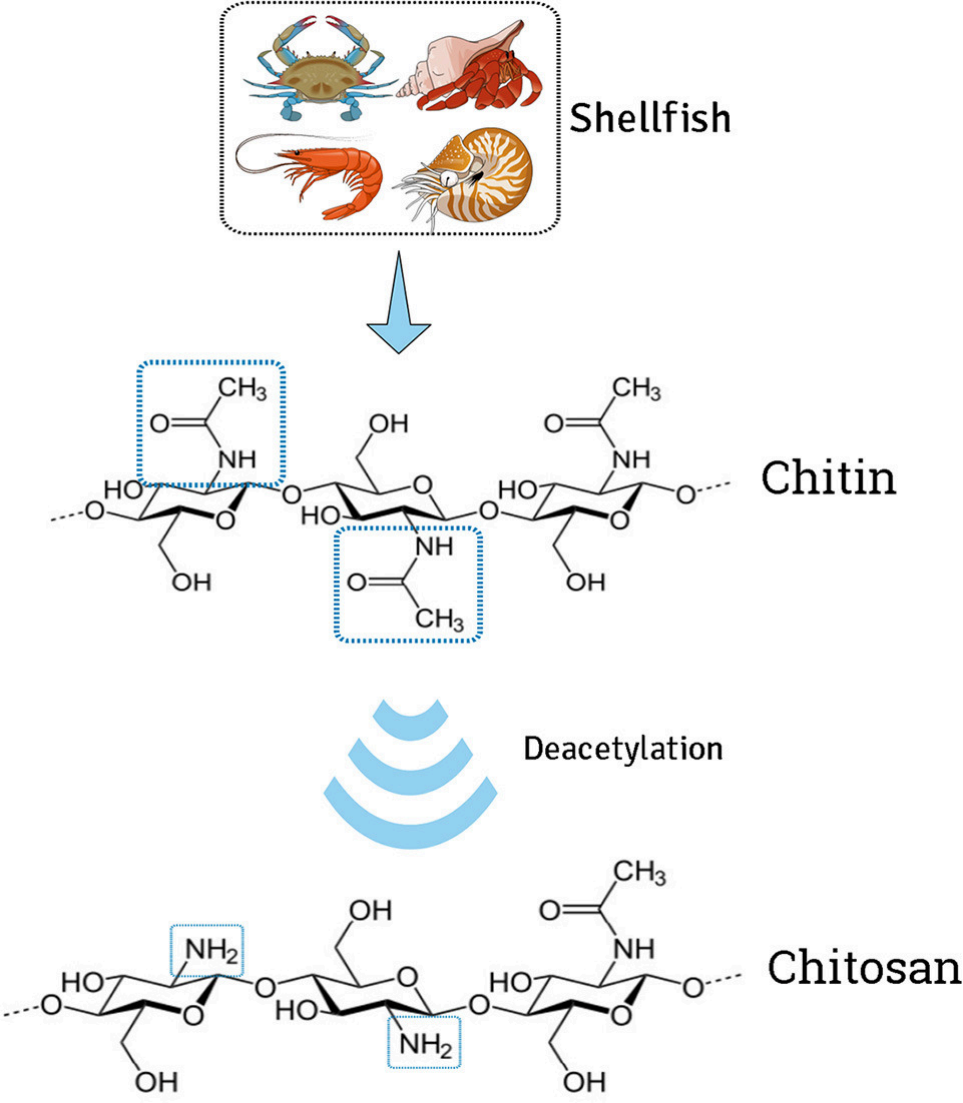
Schematic representation of the alkaline deacetylation of chitin to obtain chitosan.

**Figure 2:**
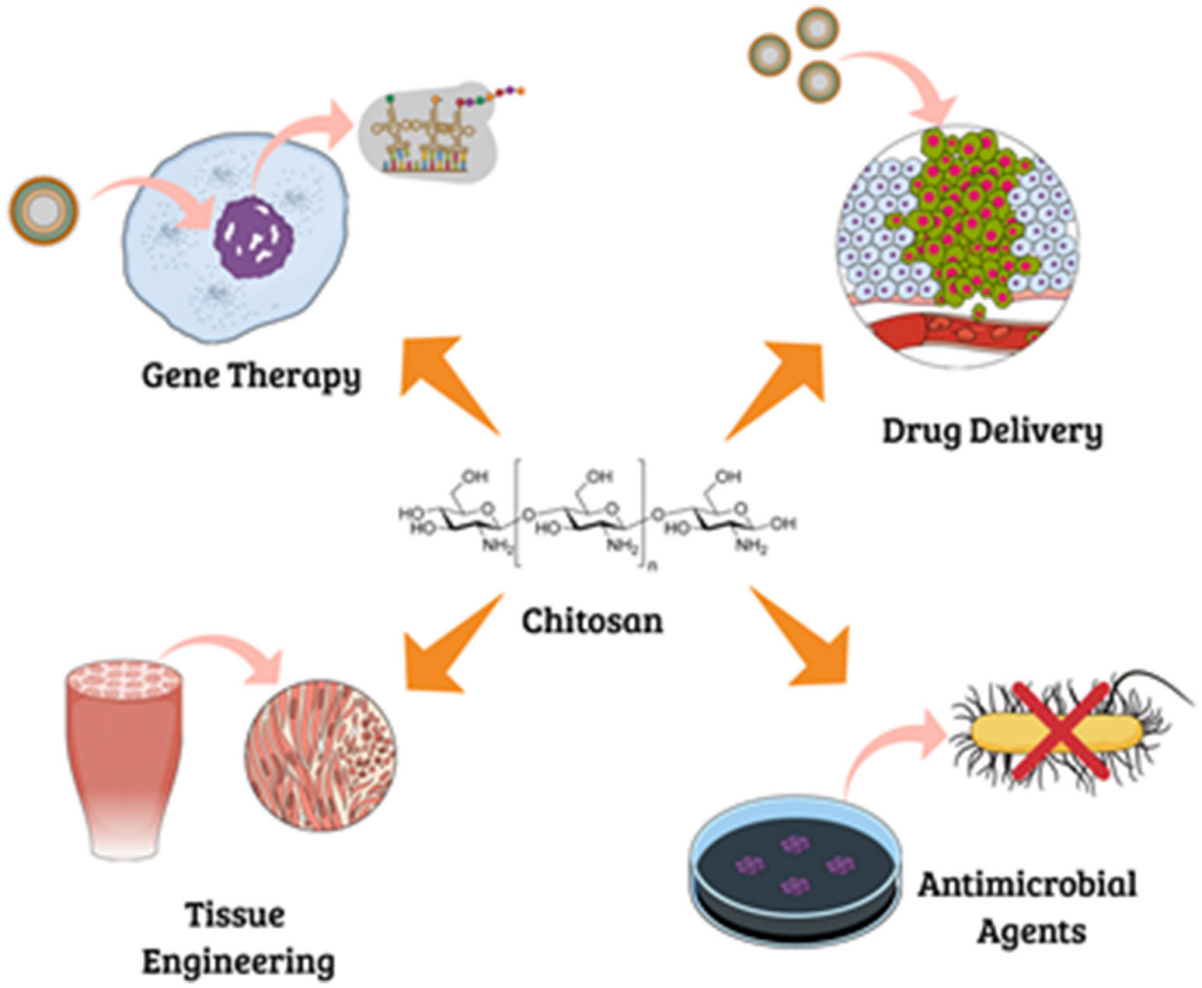
Potential applications of chitosan in the biomedical field.

Chitosan is a modified natural cationic polymer composed of β-(1→4)-linked D-glucosamine residues, obtained by partial N-deacetylation of chitin (Figure 1). This polyaminosaccharide polymer has polycationic characteristics, due to the presence of numerous amino groupings (Alves and Mano, 2008; Anitha et al., 2011), and possesses excellent chemical and biological properties, making it very attractive for applications in many areas including biology, chemistry, pharmaceuticals, medicine, agriculture, food, and environment (Yao et al., 2011; Kashyap et al., 2015). However, the amino groupings become positively charged at acid pH, so the polymer is only soluble in dilute acid solutions. Different strategies have been proposed in order to chemically modify chitosan in order to introduce new features to the polymer and extend its possible applications (Figure 2), one of which is copolymerization by grafting (Jayakumar et al., 2005; Alves and Mano, 2008; Thakur et al., 2014). Specific chemical modifications of chitosan that have been reported involve free amino groups on deacetylated units and hydroxyl groups on the C_3_ and C_6_ carbons (Filipović-Grcić et al., 2001; Prabaharan and Mano, 2006; Glasing et al., 2016).

Cyclodextrins are cyclic oligosaccharides composed of six to eight units of glucopyranosyl connected by α-(1→4) linkages (Davis and Brewster, 2004). This cyclic organization provides CDs with conical structures in which the central cavity is nonpolar, while the outer surface is polar. The characteristic cavities of cyclodextrins enable these substances to form inclusion complexes with many different kinds of molecules (Zhang et al., 2008; Nuchuchua et al., 2009; Mura, 2014). In pharmaceutical applications, CDs are extensively employed as solubilizers and stabilizers. A limiting factor is that they lack mucoadhesive properties, which can be resolved by grafting these oligosaccharides onto the structures of bioadhesive polymers such as chitosan.

Polyethylene glycol (PEG) is a hydrophilic polymer synthesized from ethylene oxide, consisting of polyether (linear or branched) terminated with hydroxyl groups (Roberts et al., 2012). PEG can be synthesized in a wide range of molecular sizes, with the different PEGs identified by a number indicating the average molecular weight (Schellekens et al., 2013). PEGs are listed by the FDA as generally safe compounds, due to their low toxicity, low immunogenicity and antigenicity, low flammability, biodegradability, and rapid excretion after administration to living organisms (CFR - Code of Federal Regulations Title 21, 2015). Due to these properties, PEGs are used in various fields, especially in the medical and pharmaceutical sectors, where conjugates of PEGs and proteins/drugs have been developed in order to extend circulation times in the blood (Garay et al., 2012). Another field of research that has emerged is the use of PEGs and their derivatives for grafting with polymers and other molecules. The high mobility of the chains, associated with conformational flexibility and water binding capability, enables the production of numerous bioconjugates (Cho et al., 2012).

Cyclodextrins grafted onto chitosan backbones have received considerable attention because carriers composed of this hybrid polymer possess the capability of CDs to form inclusion complexes with different molecules, together with the mucoadhesive properties of chitosan (Alves and Mano, 2008; Pillay et al., 2013). The grafting of PEGs onto chitosan has also been used as a strategy to improve the solubility and biocompatibility of chitosan. Generally, the terminal hydroxyl groups of PEGs are modified to generate derivatives capable of promoting nucleophilic displacements of the amino groups of chitosan. As a consequence, the grafting of PEG onto chitosan has been shown to decrease cytotoxicity and improve the stability of the resulting colloidal carrier system (Malhotra et al., 2011; Casettari et al., 2012).

**Figure 3:**
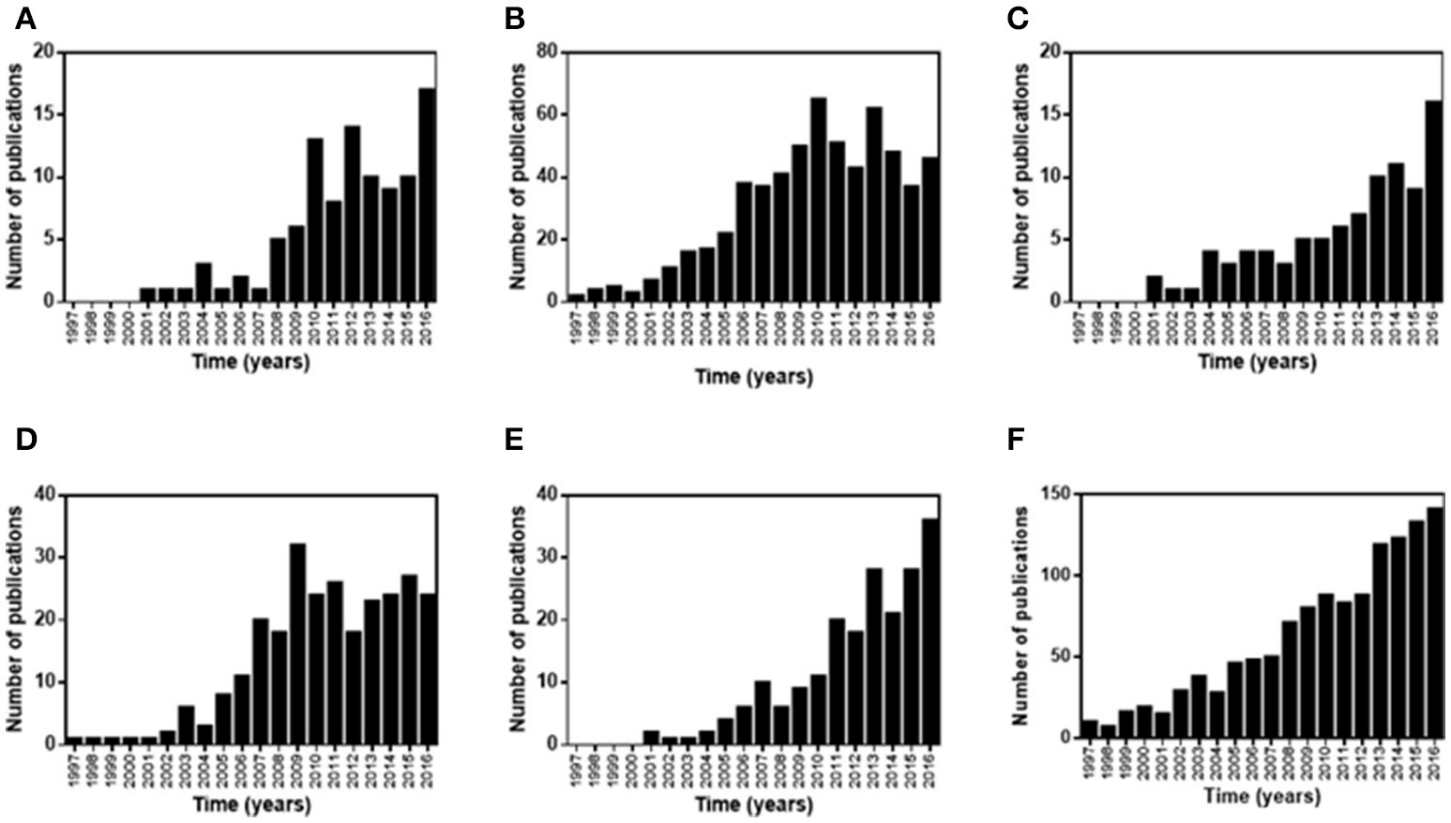
Numbers of publications during the last 20 years involving the chemical modification of chitosan. Each graph represents one combination of keywords: **(A)** Modified chitosan and pharmaceutical applications; **(B)** Chitosan and graft copolymerization; **(C)** Cyclodextrin grafted on chitosan; **(D)** Grafted poly(ethylene glycol) and chitosan; **(E)** PEGylated chitosan; **(F)** Chemical modification and chitosan.

**Figure 4:**
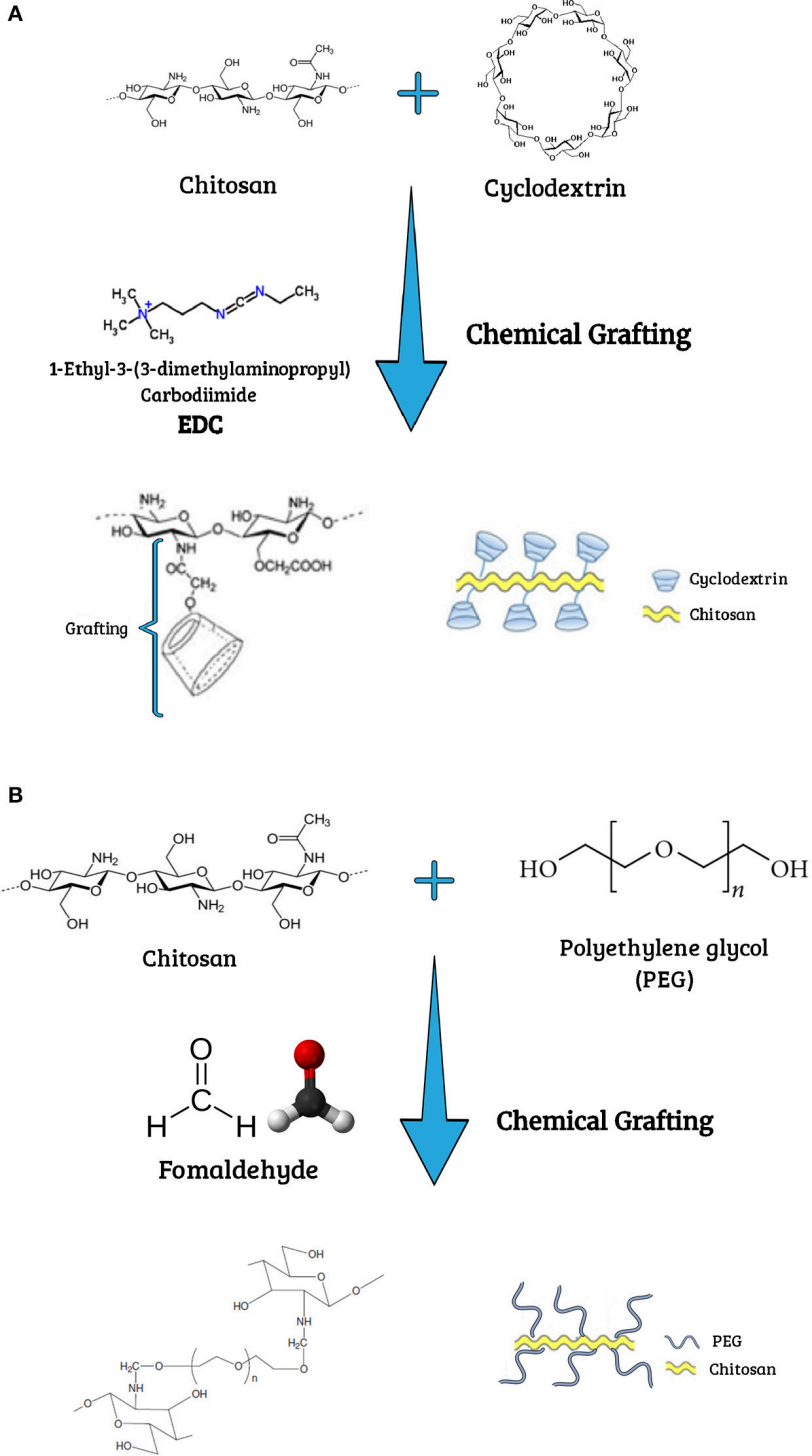
Schematic representations of **(A)** cyclodextrin chemically grafted onto chitosan, and **(B)** PEG chemically grafted onto chitosan.

In order to investigate the evolution of studies conducted during the last 20 years concerning chemical modification of chitosan, a search was performed with the ISI Web of Knowledge data base, using different combinations of keywords (cyclodextrin grafted chitosan; PEG grafted chitosan; PEGylated chitosan; modified chitosan; pharmaceutical applications). The data generated from this search are summarized in Figure 3. It can be seen that the numbers of publications in this field have increased over the years. When broad keywords were used (chemical modification and chitosan; chitosan and copolymerization), the publications increased gradually since the 1990s. For keywords with more specific terms (cyclodextrin grafted chitosan; PEG grafted chitosan; PEGylated chitosan; modified chitosan; pharmaceutical applications), increases started after the year 2000. This growth in publications reflects the interest in using hybrid polymers based on chitosan, which offer many advantages compared to pure chitosan, and indicates the relevance of research in this field due to the applicability of these polymers in many different fields. The aim of this review is to present the different types of chitosan modified with cyclodextrins or PEG (Figure 4), describing the grafting techniques and the use of these hybrid polymers in biotechnological and pharmaceutical applications.

## Grafting methods

The chemical modification of polysaccharides has received increasing attention in recent years, opening perspectives for applications of these modified macromolecules in different fields (Cumpstey, 2013; Pillay et al., 2013; Li et al., 2016). Among the modification methods, graft polymerization is a promising strategy that can be used to introduce a variety of functional groups to polymers (Bhattacharya, 2004; Jayakumar et al., 2005). In the case of chitosan, characteristics such as low water solubility, insolubility in organic solvents, and lack of thermal plasticity have limited its uses in some areas. Chemical modification can be used to overcome these problems (Sashiwa and Aiba, 2004; Olteanu, 2007; Li et al., 2016).

Grafting is a modification technique in which polymer monomers are covalently bonded to the backbone of a parent polymer (the substrate) (Bhattacharya, 2004). This process alters the surface properties, while the modified product still retains the bulk properties of the parent polymer (Yang et al., 2007). Use of grafting reduces desorption and conveys long-term chemical stability because of its covalent nature. Other techniques such as surface coating modifications do not provide temporal stability and the coatings are liable to desorption (Witono et al., 2012). There are various methods of graft copolymerization, employing a single monomer or mixtures of two or more monomers. Table 1 summarizes some of the characteristics and limitations of these different methodologies for grafting cyclodextrins onto chitosan.

**Table 1:** Summary of the different grafting methods, reaction mechanisms, characteristics, and limitations.

**Grafting method**	**Reaction mechanism**	**Main characteristics**	**Limitations**
Chemical	Generation of free radicals and/or ions by chemical products (Pillay et al., 2013)	Modifies the surface properties without changing the bulk properties (Al-Malaika, 2012)	Production of homopolymers; difficult purification; use of harmful reagents (Jayakumar et al., 2005)
Enzymatic	Generation of free radicals by enzymes (Fillat et al., 2012)	High specificity with chemical groups; synthesis of purer products; no use of harmful reagents (Jayakumar et al., 2005)	Tight control needed of pH, temperature, and enzyme concentrations (Liu et al., 2014)
Photo-initiated	Formation of free radicals by direct ultraviolet or microwave irradiation (Deng et al., 2009)	Direct generation; grafting process with or without a sensitizer; no need for washing procedures (Wang et al., 2010)	Only surface modification; irradiation is not penetrative (Wang et al., 2010)
Radiation	Generation of free radicals and/or ions by gamma/alpha radiation (Lv et al., 2013)	No need for an initiator; acts directly on the polymer backbone; greater penetration power; grafting at different depths of the polymer matrix (Yamaki et al., 2003)	More expensive than other techniques; can damage the polymer, causing degradation and/or decomposition (Chen et al., 2002)

### Grafting by schiff base formation and reductive amination

**Scheme 1:**
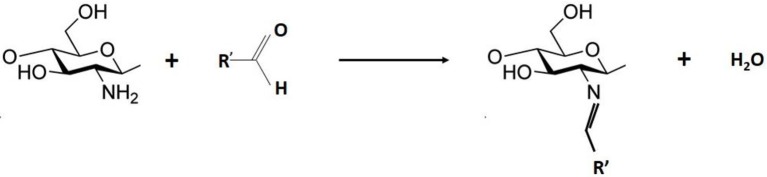
General mechanism of imine formation.

Functional groups containing a nitrogen-carbon double bond (C = N), where nitrogen forms a bond with aryl or alkyl groups, are known as Schiff bases. The Schiff base reaction (Scheme 1), first described by Schiff (1864), can proceed by a condensation reaction of an aldehyde or ketone with a primary amine (Schiff, 1864; da Silva et al., 2011; Ciaccia and Di Stefano, 2015). In order to favor the reaction, the solution must be sufficiently acid, resulting in protonation of the carbonyl compound and making the carbon more susceptible to nucleophilic attack (da Silva et al., 2011; Ciaccia and Di Stefano, 2015).

**Scheme 2:**
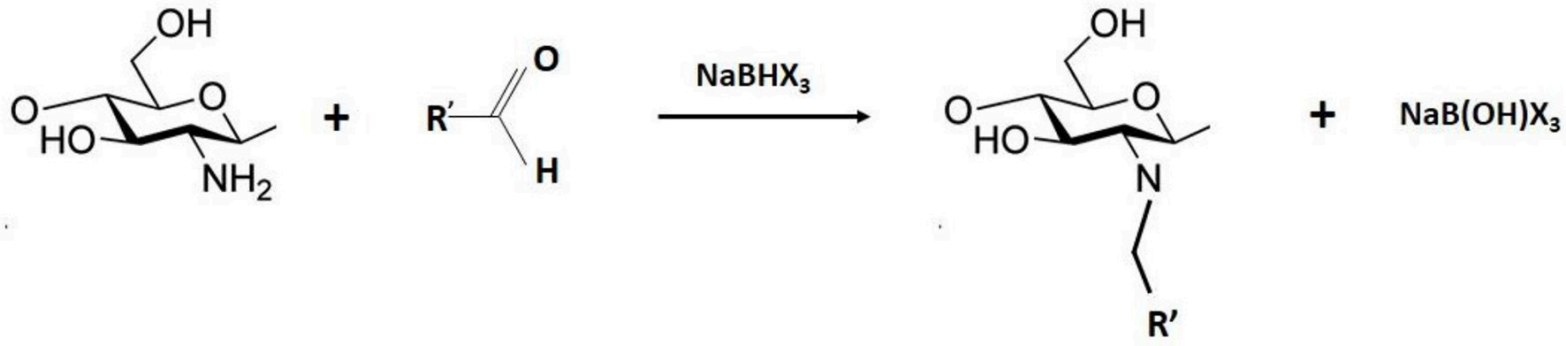
General mechanism of reductive amination.

Substituent groups can be inserted into chitosan backbones by reductive amination (Scheme 2), which is a variation of Schiff base formation (Baxter and Reitz, 2002; Goszczynska et al., 2015). However, in this case, chitosan reacts with an aldehyde or ketone, resulting in an imine intermediate that is subsequently converted to an N-alkyl or N-aryl derivative by reduction employing sodium borohydride (NaBH_4_) or sodium cyanoborohydride (NaCNBH_3_) (Baxter and Reitz, 2002). The amines generated by these reactions are stable toward hydrolysis, and other advantages of this route are selective functionalization of the amino groups of chitosan and a homogeneous distribution of substituent groups in the chitosan backbone (Sajomsang, 2010; Badawy and Rabea, 2013).

Du and Hsieh (2007) performed PEGylation of chitosan by reductive amination and acylation, resulting in PEG-N-chitosan and PEG-N,O-chitosan, respectively. Chitosans with different molecular weights (137, 190, and 400 kDa) were used, together with different molecular weight PEGs (550, 2,000, and 5,000 Da). Both reductive amination and acylation resulted in the conversion of hydroxyl-terminal groups of PEG to more reactive groups. It was found that in both reactions, shorter chain lengths of either chitosan or PEG resulted in increased PEGylation. The degree of substitution of PEG-N-CS was between 0.12 and 0.44, while for PEG-N,O-CS, values of 1.50 and 0.60 were obtained for chitosan functionalized with 550 and 2,000 Da PEGs, respectively. All the PEGylated materials became water-soluble, due to the high content of PEG. The authors reported that DNA condensation was much more effective when mediated by pyrene or multi-walled carbon nanotubes.

Liu et al. (2008) prepared chitosan modified by β-cyclodextrin using a Schiff base reaction between 6-O-(4-formylphenyl)-β-cyclodextrin and chitosan. The modified chitosan was used to construct different supramolecular aggregations with pyrene, multi-walled carbon nanotubes, and a combination of pyrene and multi-walled carbon nanotubes. The effect of these supramolecular aggregates on DNA condensation was also evaluated.

### Grafting by amide formation

**Scheme 3:**
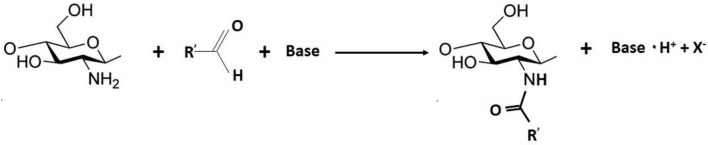
General mechanism of amide formation.

Amide bonds are usually formed from pre-activated carboxylic acid derivatives such as acid chlorides and anhydrides, or by using diimide derivatives (Montalbetti and Falque, 2005; Pattabiraman and Bode, 2011). This reaction (Scheme 3) can proceed in different ways: (i) formation of an intermediate acylating agent, followed by aminolysis; (ii) formation of a reactive acylating agent from the acid, followed by treatment with amine; and (iii) addition of an activating or coupling agent, resulting in the *in situ* generation of an acylating agent from the acid, in the presence of imine (Montalbetti and Falque, 2005; Pattabiraman and Bode, 2011). Carbodiimides have shown great potential for the attachment of molecules onto the chitosan backbone, due to their allene functional groups (Mourya and Inamdar, 2008). The compound 1-ethyl-3-(3-(dimethylamino)propyl) carbodiimide (EDC) is the most widely used carbodiimide, because of its aqueous solubility (Hermanson, 2013). However, there are other carbodiimides that can be used, such as dicyclohexylcarbodiimide (DCC) and N,N′-diisopropylcarbodiimide (DIC), which require organic solvents since they are water-insoluble. The transformation of EDC into a non-toxic urea derivative results in low toxicity associated with the reactions performed with this compound (Hermanson, 2013).

Lin and Hsu (2015) prepared galactosylated chitosan grafted with methoxy poly(ethylene glycol) (mPEG) or short chain poly(ethylene glycol) diacid (PEGd), under aqueous conditions, by amide formation employing EDC, involving the amino groups of chitosan and the carboxylic groups of PEG. The degree of substitution of PEG on the chitosan was 3.7%. Evaluation was also made of the formation of a polyplex with DNA. Chitosan grafted with mPEG formed the most stable polyplex with DNA, followed by galactosylated chitosan grafted with PEGd. Compared to naked DNA, the polyplex enhanced DNA cellular transfection in HepG2 cells.

Tan et al. (2012) prepared and characterized chitosan grafted with carboxymethyl-β-cyclodextrin (CM-β-CD) for subsequent application in anticancer drug delivery. The grafting employed carbodiimide (EDC), which promoted amide formation between the carboxylic groups of CM-β-CD and the amine groups of chitosan. The degree of substitution was measured by a colorimetric method and showed that there was about one grafted CM-β-CD on every fifteenth monosaccharide unit of chitosan (223.05 μmol g^−1^). The carrier was able to release the anticancer drug at pH 5.0, but not at pH 7.4. This carrier could be used for anticancer drug release because it was pH-sensitive.

### Grafting by “click reactions”

**Scheme 4:**
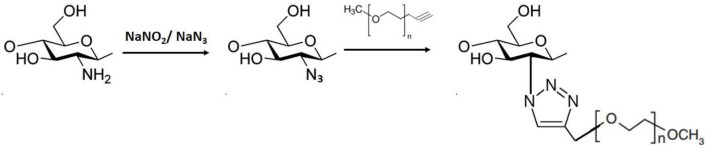
1,3-dipolar cycloaddition (adapted from Kulbokaite et al., 2009).

Although traditional organic syntheses are important tools for the production of new materials, they are inevitably time consuming and expensive (Nwe and Brechbiel, 2009). In order to overcome these limitations, Kolb et al. (2001) introduced the concept of click chemistry, establishing a set of criteria to encourage simplicity and robustness in chemical syntheses. They described a set of reactions that fulfilled these criteria, including chemoselective reactions with high yield, broad scope, removal of the product without use of chromatography, simplicity, and the ability to operate under different conditions. Any reaction fulfilling these prerequisites could be considered a click reaction. The term “click chemistry” refers to reactions where two functional groups react exclusively with each other and which can be performed at room and physiological temperatures. These reactions are divided into four major groups: (i) cycloadditions of unsaturated species; (ii) nucleophilic ring-openings; (iii) non-aldol type carbonyl chemistry; and (iv) additions to carbon-carbon multiple bonds (Hein et al., 2008). Among all the click reactions (Scheme 4), copper-catalyzed Huisgen 1,3-dipolar cycloadditions (CuAAC) of alkynes and azides are the most used (Wallyn et al., 2011; Kim and Kim, 2014).

Kulbokaite et al. (2009) studied the preparation and characterization of methoxy PEGylated chitosan (chitosan-N-MPEG) copolymers using click reactions. The polymer was synthesized by the reaction of N-azidated (azide groups) chitosan with acetylene-terminated mPEG (alkyne groups), using 1,3-dipolar cycloaddition. The reaction was successful in a medium consisting of water and methylene chloride (1:1 v/v), but failed in a solution of 5% LiCl in N-methyl-2-pyrrolidone. The failure was attributed to the high concentration of Li^I^, which resulted in coordination competition with Cu^I^ and decreased its catalytic activity. It was shown that an equimolar ratio of CS and mPEG, or even low concentrations of mPEG, resulted in unreactivity of the alkyne groups of mPEG and consequently low degrees of substitution. Polymers synthesized with excess amounts of alkyne groups from mPEG showed degrees of substitution up to 40%, solubility in water, and residual amounts of Cu.

Chen et al. (2013) synthesized chitosan 6-OH immobilized cyclodextrin by click reaction. Firstly, the amino group of chitosan was protected by Schiff base formation with benzaldehyde, followed by the generation of C_6_-OH p-toluenesulfonate. Subsequently, nucleophilic substitution of sulfonate with NaN_3_ resulted in Schiff base protected chitosan (BCTS-6-N_3_). Separately, alkynylic β-cyclodextrin (CD-OPg) was obtained. After the production of these substrates, the Cu (I)-catalyzed click reaction between BCTS-6-N_3_ and CD-OPg resulted in the 2-benzadehylde Schiff base chitosan 6-OH immobilized cyclodextrin derivative (BCTS-6-CD). The removal of the protecting group then resulted in CTS-6-CD. The loading of immobilized cyclodextrin in the final product was 223.17 μmol g^−1^.

### Grafting by nucleophilic substitution reaction

**Scheme 5:**
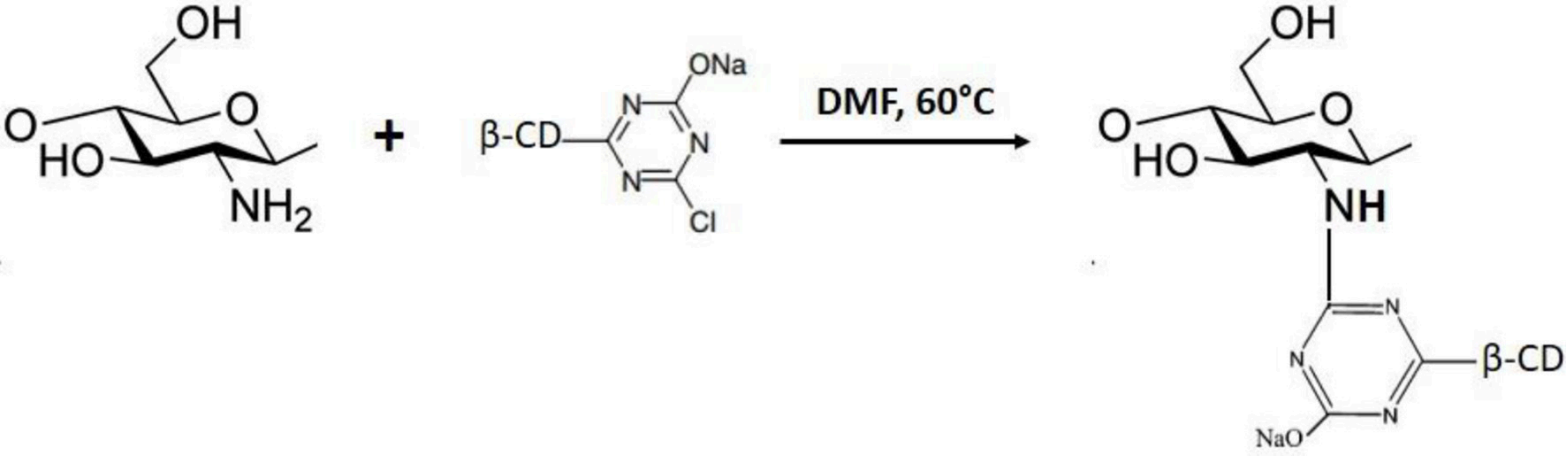
Nucleophilic substitution reaction (adapted from Martel et al., 2001).

In addition to the two hydroxyl groups (primary and secondary), chitosan has a third reactive site that is attractive for chemical modification: the primary amine group (Kyzas and Bikiaris, 2015). The non-bonding electron pair on the primary unit acts as a proton acceptor, making chitosan a potent nucleophile (Scheme 5) (Buschmann et al., 2013). Tosyl groups are well known in organic chemistry because they are good electrophiles and leaving groups. Tosylation of the 6-OH group of chitosan has been employed in order to produce tosyl-chitosan as an intermediate, prior to further chitosan modification. Chen et al. (2012) prepared chitin with tosylated 6-OH groups, which were displaced by a monoamino β-cyclodextrin derivative via nucleophilic substitution. Subsequently, the cyclodextrin-grafted chitin was deacetylated, producing the modified chitosan. This method was effective for the production of 6-OH substituted cyclodextrin derivatives of chitosan with high levels of substitution, where the final polymer showed a substitution capacity of around 128.68 mol g^−1^.

Monochlorotriazinyl groups are known in organic chemistry for their capacity to form covalent bonds with nucleophilic groups. Martel et al. (2001) prepared a chitosan derivative by attaching a monochlorotriazinyl derivative of β-CD onto the chitosan backbone. This reaction occurred by nucleophilic substitution of the chloride atoms of the β-CD by the amino groups of chitosan. The high degree of substitution (2.8) resulted in an insoluble product.

### Grafting by photo-initiation

Photo-initiated grafting is a simple process based on the formation of radicals by the application of electromagnetic radiation (ultraviolet or microwave). The technique can follow two different routes, either with or without a sensitizer such as methyl methacrylate (Muftuoglu et al., 2004). When the polymer is non-photolabile, the use of a sensitizer is required, with the molecule absorbing light and attaining an excited/high energy state. The sensitizer then transfers this energy to the polymer, causing dissociation of the molecule and formation of the free radicals responsible for the grafting process (Deng et al., 2009). The main photosensitizers include benzoin ethyl ether, acrylated azo dyes, aromatic ketones (benzophenone and xanthone), and metal ions. However, there is still a need for photosensitizers that are more efficient (Deng et al., 2009; Wang et al., 2010).

Sharma and Rajesh (2017) reported a fast way to prepare β-cyclodextrin-grafted chitosan by a microwave assisted method. The capability of the resultant polymer to adsorb palladium (II) was evaluated. The method employing the microwave reactor required only 5 min at 140°C to complete the reaction, whereas the conventional method necessitated approximately 6 h of stirring at 70°C. The polymers showed high selectivity toward palladium (II), with around 96% being recovered from a contaminated solution.

Graft polymerization of chitosan can also be initiated by gamma radiation and enzymes (Wang et al., 2007; Karaki et al., 2016; Li et al., 2016; Yang et al., 2016). It can therefore be seen that this is a useful technique for improving the properties of chitosan and broadening the range of its possible applications. Selection of the graft copolymerization method to be used can be based on the intended use of the hybrid polymer, and each of the different methods has variables that can influence the grafting process (Sun et al., 2003; Thakur et al., 2014).

## Characterization of grafted polymers

There has been rapid development in the field of grafting copolymerization of polysaccharides in the last decades, because grafted polysaccharides present new characteristics that strongly influence the properties of the material and open up new potential applications (Harish Prashanth and Tharanathan, 2007; Ji et al., 2014; Kyzas and Bikiaris, 2015). The characterization of grafted polysaccharides is essential in graft copolymerization research. There are several techniques employed in polymer characterization, which are selected according to the properties studied (Ratner, 1980; Sapsford et al., 2011; Meléndez-Ortiz and Bucio, 2015). Techniques used to characterize graft copolymerization (Table 2) can be divided into six main areas: (i) graft properties (such as the degree of substitution/graft); (ii) thermodynamic measurements; (iii) surface chemistry; (iv) surface topography; (v) crystalline structure; and (vi) mechanical properties (Ratner, 1980; Meléndez-Ortiz and Bucio, 2015).

**Table 2:** Main techniques employed to characterize graft copolymerization.

**Technique**	**Applications**	**References**
Nuclear magnetic resonance spectroscopy (2D NMR, NOE NMR, TROSY NMR, and DOSY NMR)	NMR spectroscopy is a reliable and comprehensive technique widely used in polymer science. Rapid developments in NMR technology have led to many applications, mainly based on through-bond interactions, through-space interactions, chemical exchange, and molecular self-diffusion	Bhattarai et al., 2005; Malhotra et al., 2011, 2013; Novoa-Carballal et al., 2013; Hassani Najafabadi et al., 2014; Tsao et al., 2015; Jing et al., 2017
Infrared spectroscopy Fourier transform IR (FT-IR) and attenuated total reflection IR (ATR-IR)	The IR technique is frequently used for the characterization of functionalized polymers by the detection of specific spectral bands. The principle of the technique is based on detection of molecular stretching and bending vibration modes following the absorption of IR radiation by the sample	Kolhe and Kannan, 2003; Papadimitriou et al., 2012; Hassani Najafabadi et al., 2014; Deygen and Kudryashova, 2016; Sahariah et al., 2016
UV spectroscopy	Ultraviolet/visible (UV-Vis) spectroscopy is also very useful for polymer characterization. The technique involves the absorption of electromagnetic radiation (200–800 nm), with the absorption by some organic molecules being restricted to certain functional groups. This enables investigation of the transfer of electrons between orbitals or bands of atoms, ions, and molecules. For better characterization, the technique is commonly used in conjunction with FTIR	Chan et al., 2007; Ho et al., 2015; Liu et al., 2017
Raman spectroscopy Resonance Raman (RRS), surface enhanced Raman (SERS), and surface enhanced resonance Raman spectroscopy (SERRS)	The Raman spectroscopy technique is used for the characterization of functionalized polymers, often as a complement to infrared analysis. It is based on the inelastic dispersion of monochromatic radiation, resulting in fingerprint Raman bands	Jokerst et al., 2011; Zajac et al., 2015; Dubey and Gopinath, 2016
X-ray diffraction	X-ray diffraction (XRD) is commonly used for the characterization of various polymers and bioconjugates, providing important information about the structures of crystalline samples. Analysis of the polymorphism of such compounds is important because it is directly related to their application properties	Zhang et al., 2014; Du et al., 2016; Esmaeili and Ghobadianpour, 2016
High performance liquid chromatography (HPLC) Reverse phase (RPC), ion exchange (IEC), and size exclusion chromatography (SEC)	HPLC techniques are widely used in purification processes and for polymer characterization, especially evaluation of molecular weight, since this property directly influences the biological and physicochemical properties of the polymer	Casettari et al., 2012; Zhang et al., 2013
Mass spectroscopy (MS)	MS is a destructive analytical technique used to analyze samples based on their mass-to-charge ratio, obtaining information such as molecular mass, molecular structure, and purity	Chan et al., 2007; Weidner and Trimpin, 2010; Zu et al., 2017
Thermogravimetric analysis (TGA)	TGA is an important method for the characterization of polymers. The change in sample mass according to temperature can provide evidence of the functionalization processes. The technique uses a high-precision balance to measure changes in mass	Davidovich-Pinhas et al., 2014; Hassani Najafabadi et al., 2014; Hauptstein et al., 2014; Najafabadi et al., 2014; Garcia-Valdez et al., 2015; Darabi et al., 2016
Differential scanning calorimetry (DSC)	Another important thermally based technique is exploratory differential calorimetry. In the characterization of polymers, the technique allows evaluation of different transition stages, including fusion, glass transition, crystallization, and decomposition. The technique is complementary to TGA and can provide stability and structural information	Mao et al., 2005; Najafabadi et al., 2014; Huang et al., 2015
Electron microscopy Transmission electron microscopy (TEM), scanning electron microscopy (SEM)	Electron microscopy techniques are commonly used to investigate morphological and structural differences in functionalized polymers. The sample is irradiated with an electron beam and detection is performed in transmission or reflectance mode, after which an image is generated	Prego et al., 2006; Hassani Najafabadi et al., 2014; Xie et al., 2014; Lin and Hsu, 2015; Luo et al., 2016

## Applications

This topic will be divided into two sections: (i) Examples of hybrid copolymers composed of cyclodextrin and chitosan (CD-CS) that have been produced in order to improve the solubility of the associated compound, and their main applications; (ii) discussion of changes in the solubility of copolymers composed of polyethylene glycol grafted onto chitosan (CS-PEG), and their applications in different fields.

### Cyclodextrin-grafted chitosan

Asamoah-Asare et al. (2014) studied the preparation and characterization of nanoparticles composed of carboxymethyl chitosan (CMC) and carboxymethyl β-cyclodextrin (CM-β-CD), in order to produce a carrier system capable of carrying and releasing both hydrophilic and hydrophobic drugs. Investigation of the feasibility of producing the nanoparticles employed different concentrations of CMC (0.05–1.50%, w/v), while maintaining a constant CM-β-CD concentration (0.8 mg/mL). The results showed that use of a low concentration of CMC produced large nanoparticles, while a high concentration of CMC produced small nanoparticles. In addition, it was observed that use of the lowest and highest CMC concentrations did not result in production of sufficient nanoparticles to be characterized. All the nanoparticles showed negative surface charge, and the zeta potential of the nanoparticles increased as the CMC concentration decreased. The nanoparticles produced were very promising as carriers for both hydrophilic and hydrophobic drugs.

In another example involving drug delivery, Daimon et al. (2014) studied the preparation and physicochemical properties of β-CD grafted onto chitosan in order to develop a possible carrier system for drugs, employing insulin as a model compound. Investigation was made of the supramolecular aggregate formation mechanism and the morphological effects of the action of ionic species. The results revealed different morphologies of the macromolecular aggregates, depending on the media conditions employed. The macromolecular structure, nanoparticles, and large aggregates were formed in acetate, citrate, and phosphate buffers, respectively. In order to simulate the environment after oral administration, the network structure formed in buffer solution was diluted with phosphate buffer and it was observed that the network was maintained. Multivalent interactions consisting of host-guest and electrostatic interactions between β-CD-grafted chitosan and insulin enabled strong binding over a wide pH range.

In another study, Kono and Teshirogi (2015) reported the preparation of smart hydrogels for use as carrier systems for drugs, consisting of β-CD-grafted CMC hydrogels with different ratios of CMC and CD (CD-g-CMCs), produced using 1-ethyl-3-(3-dimethylaminopropyl) carbodiimide (EDC) and N-hydroxysuccinimide (NHS) as the coupling method. One hydrogel was prepared containing only carboxymethyl chitosan, without addition of the CD, and CD-g-CMC gels were prepared with different CD concentrations. Evaluation was made of the swelling at pH 4, 7, and 10, as well as drug adsorption/absorption and release. The drug model employed was acetylsalicylic acid (aspirin). The hydrogels showed low swelling at pH 4 and similar absorptions at pH 7 and 10, indicating that charged groups in the hydrogels participated in the swelling mechanism. The uptake of acetylsalicylic acid by the CD-g-CMC hydrogels was strongly dependent on the amount of CD grafted. After 24 h, the CD-g-CMC hydrogels adsorbed 23.9 μmol.g^−1^, while the CMC hydrogel only adsorbed 2.4 μmol.g^−1^ during the same period. The CD-g-CMC hydrogels showed fast release of acetylsalicylic acid in the first 2 h, after which the release became slower. The CMC hydrogels showed more rapid release, with the majority of the drug (86%) released within 2 h.

Other potential biomedical applications of systems based on cyclodextrins grafted onto chitosan that have been reported in the literature are summarized in Table 3.

**Table 3:** Development of systems composed of cyclodextrin grafted onto a chitosan backbone for applications in different biomedical areas.

**Type of chitosan**	**Type of CD**	**Spacer between CS and CD**	**Type of reaction**	**Crosslinking agent or reaction initiator**	**References**
Thiolated chitosan	Carboxymethyl-β-cyclodextrin	Amine bonds	Amide formation	Hexamethylene diisocyanate	Alamdarnejad et al., 2013
Chitosan	β-cyclodextrin	Imine bonds	Schiff base formation	^*^	Anirudhan et al., 2013
Chitosan	β-cyclodextrin citrate	^*^		Citric acid/ Formic acidgo	Eltahlawy et al., 2006
Chitosan	O-p-toluenesulfonyl-β-cyclodextrin	Imine bonds	Nucleophilic displacement	Tosyl groups	Gonil et al., 2011
Carboxymethyl chitosan	Carboxymethyl-β-cyclodextrin	Amide bonds	Amide formation	1-Ethyl-3-(3-dimethylaminopropyl) carbodiimide (EDC) and N-hydroxysuccinimide (NHS)	Prabaharan and Gong, 2008
Chitosan	O-p-toluenesulfonyl-β-cyclodextrin	Imine bonds	Nucleophilic displacement	Tosyl groups	Sajomsang et al., 2012
Chitosan	O-p-toluenesulfonyl-β-cyclodextrin	Imine bonds	Nucleophilic displacement	Tosyl groups	Yuan et al., 2013
Chitosan	Carboxymethyl-β-cyclodextrin	Amide bonds	Amide formation	1-Ethyl-3-(3-dimethylaminopropyl) carbodiimide (EDC) and N-hydroxysuccinimide (NHS)	Prabaharan and Jayakumar, 2009
Chitosan	Polyethylenimine β-cyclodextrin	Imine bonds	Reductive amination	Tosyl groups	Ping et al., 2011
Chitosan	Carboxymethyl-β-cyclodextrin	Amide bonds	Amide formation	1-Ethyl-3-(3-dimethylaminopropyl) carbodiimide (EDC) and N-hydroxysuccinimide (NHS)	Song et al., 2017
Chitosan	β-cyclodextrin	^*^	1, 3-dipolar cycloaddition	Cu (I)	Lu et al., 2014
N-maleoyl chitosan	β-cyclodextrin	^*^	Nucleophilic substitution	Maleoyl group	Hou et al., in press

* *uninformed*.

It can be seen from Table 2 that there are various potential applications of cyclodextrin-grafted chitosan systems. However, further advances are required in order to make it viable to manufacture these products at the commercial scale. Some of the synthesis procedures will need to be optimized in order to produce these grafted compounds for use in commercial products and industrial processes.

### Chitosan-PEG

This section describes examples of the use of PEG grafted onto chitosan, considering the potential advantages of this strategy.

In a recent study by Anraku et al. (2014), chitosan was modified with PEG to increase the biocompatibility and water solubility of the polysaccharide. The formaldehyde linking method was used to modify chitosan, using different molecular weights of chitosan (22, 38, and 52 kDa) and a derivative of polyethylene glycol. Nanoparticle aggregates of PEG-CS were also prepared, and the functional and structural properties of the materials were investigated at neutral pH. The copolymer obtained possessed a cationic main chain composed of chitosan, and a non-ionic hydrophilic chain containing PEG. The degrees of substitution of the different copolymers synthesized were 25.6, 28.9, and 27.2 mol% for the mPEG-CS prepared with 22, 38, and 53 kDa chitosan, respectively. The mPEG-CS22, mPEG-CS38, and mPEG-CS53 particle sizes were 259, 413, and 452 nm, respectively, and all the copolymers exhibited almost identical zeta potentials (around +31 mV). The scavenging activities for 2,2-diphenyl-1-picrylhydrazyl (DPPH) revealed that the smallest nanoparticles had the greatest antioxidant activity.

Fu et al. (2014) reported the preparation and characterization of methoxypolyethylene glycol (mPEG) grafted onto chitosan (mPEG-g-CS) and the subsequent preparation of self-assembled polymeric micelles by the ultrasonic method. The integrity of the micelles was evaluated under different pH conditions, using 5-fluorouracil (5-FU) as a model drug. The solution pH affected the solubility of the copolymer, which increased slightly between pH 4 and 5. Micelles were not formed at solution pH greater than 7 or less than 4, and a higher pH favored larger particles. The micelles presented spherical morphology, without aggregation, and particle sizes ranged between 150 and 200 nm. The release profile showed a burst effect during the initial 0.5 h, followed by delayed release.

In another example, Hassani Najafabadi et al. (2014) prepared a PEGylated chitosan by conjugating methoxypolyethylene glycol to the hydroxyl group of chitosan. For this, the most reactive groups (NH_2_) were first protected with a surfactant. The resulting copolymer was used to prepare nanoparticles loaded with ibuprofen. Dynamic light scattering measurements showed that the loaded nanoparticles presented a mean size distribution of around 80.6 nm, while AFM, TEM, and SEM analyses revealed that the nanoparticles were spherical. The encapsulation efficiency was dependent on the amount of chitosan-PEG, with an increase in the chitosan-PEG amount from 0.1 to 1% (w/v) increasing the ibuprofen encapsulation efficiency from 41 to 99%. When only chitosan was employed in the drug release experiments, the drug was completely released in 12 h. When the copolymer (chitosan-PEG) was employed, release of ibuprofen from the nanoparticles remained incomplete after 48 h. Both chitosan and the copolymer showed near-first order drug release.

Other examples of applications of PEG grafted onto chitosan are listed in Table 4.

**Table 4:** Development of systems prepared with poly(ethylene glycol) grafted onto a chitosan backbone for applications in different biomedical areas.

**Type of chitosan**	**Type of PEG**	**Spacer between CS and PEG**	**Type of reaction**	**Crosslinking agent or reaction initiator**	**References**
Chitosan	Methoxy poly(ethylene glycol)	Amine bonds	Schiff base formation/reductive amination	Aldehyde groups	Bhattarai et al., 2005
Carboxymethyl chitosan	Poly(ethylene glycol) monoacrylate	Amide bonds	Nucleophilic substitution	2, 2 dimethoxy-2-phenyl acetophenone	El-Sherbiny and Smyth, 2010
Chitosan	Methoxy poly(ethylene glycol)	Amide bonds	Nucleophilic Michael-type reaction	Aldehyde groups	Han et al., 2011
Chitosan	Dihydroxy poly(ethylene glycol)	Amine bonds	Reductive amination	Sodium naphthalene	Ito et al., 2013
Chitosan	Methoxy poly(ethylene glycol)	Amide bonds	Amide formation	4-dicyclohexylcarbodiimide and N-hydroxysuccimide	Jeong et al., 2008
Carboxymethyl chitosan	Methoxy poly(ethylene glycol)	Amide bonds	Amide formation	1-ethyl-3-(3-dimethylaminopropyl) carbodiimide hydrochloride	Jeong et al., 2010
Chitosan	Galactosylated poly(ethylene glycol)	Amide bonds	Amide formation	1-Ethyl-3-(3-dimethylaminopropyl) carbodiimide (EDC) and N-hydroxysuccinimide (NHS)	Jiang et al., 2008
Chitosan	Methoxy poly(ethylene glycol)	Amide bonds	Amide formation	4-dimethylaminopyridine and 1-ethyl-3-(3-dimethylaminopropyl) carbodiimide hydrochloride (EDC.HCl)	Liang et al., 2011
Chitosan	Monomethylated poly(ethylene glycol)	Amine bonds	Reductive amination	Aldehyde groups	Papadimitriou et al., 2012
Chitosan	Poly(ethylene glycol)	Amide bonds	Amide formation	N-hydroxysuccinamide and [N-(3-dimethylaminopropyl)-N'- ethylcarbodiimide hydrochloride]	Prego et al., 2006
Chitosan	Poly(ethylene glycol)	Amine bonds	Schiff base formation/reductive amination	Aldehyde groups	Zhang, 2008
Chitosan	Poly(ethylene glycol)	Amide bonds	Amide formation	1-Ethyl-3-(3-dimethylaminopropyl) carbodiimide (EDC) and N-hydroxysuccinimide (NHS)	Sharma et al., 2017
Chitosan oligosaccharide	Methoxy poly(ethylene glycol) succinimidyl succinate	Amide bonds	Amide formation	1-Ethyl-3-(3-dimethylaminopropyl) carbodiimide (EDC) and N-hydroxysuccinimide (NHS)	Termsarasab et al., 2014
Chitosan	Poly(ethylene glycol)	Amide bonds	Amide formation	1-Ethyl-3-(3-dimethylaminopropyl) carbodiimide (EDC) and N-hydroxysuccinimide (NHS)	Prego et al., 2006

The findings reported in the literature indicate that the strategy of grafting PEG onto chitosan is a good option for extending the applications of chitosan in different biomedical areas. However, synthesis procedures need to be developed at the industrial scale in order to enable the use of these materials in biotechnological and pharmaceutical applications, including commercial products and industrial processes.

## Conclusions and perspectives

Chitosans are aminopolysaccharides whose structures afford excellent possibilities for chemical alteration. Modifications of the chitosan skeleton are made in order to impart suitable properties and functionalities for application of these substances in different areas, especially the biomedical field. Although chitosan presents attractive properties for such uses, its aqueous insolubility is one of the main factors limiting its application on a large scale. Efforts to solve this problem have involved functionalization of the polymer with molecules that improve solubility in water.

This review presents recent advances in the use of chitosan derivatives functionalized with cyclodextrins or PEG. It is clear that many studies have achieved excellent results in terms of the solubilization of hydrophobic compounds (using cyclodextrins), as well as increased solubilization of chitosan (using PEG). There is good evidence that the use of these combined systems should bring benefits in biotechnological and pharmaceutical applications. Nonetheless, synthesis processes need to be improved to enable industrial-scale production of these materials, and emphasis should be given to synthesis routes based on the concepts of green chemistry. Considerable further progress in this area will be required in order to ensure that the promising results already reported in the literature lead to viable products and processes that are able to solve problems in the areas of health and biotechnology.

## Author contributions

EC and JO wrote the manuscript and LF contributed to the discussions and revised the manuscript. All authors approved the final manuscript.

### Conflict of interest statement

The authors declare that the research was conducted in the absence of any commercial or financial relationships that could be construed as a potential conflict of interest.
